# Influence of a Passenger Position Seating on Recline Seat on a Head Injury during a Frontal Crash

**DOI:** 10.3390/s22052003

**Published:** 2022-03-04

**Authors:** Aleksander Górniak, Jędrzej Matla, Wanda Górniak, Monika Magdziak-Tokłowicz, Konrad Krakowian, Maciej Zawiślak, Radosław Włostowski, Jacek Cebula

**Affiliations:** 1 Laboratory of Vehicle Dynamics and Safety, Department of Automotive Engineering, Mechanical Faculty, Wrocław University of Science and Technology, Na Grobli 13, 50-421 Wrocław, Poland; jedrzej.matla@pwr.edu.pl (J.M.); wanda.gorniak@pwr.edu.pl (W.G.); monika.magdziak-toklowicz@pwr.edu.pl (M.M.-T.); 2 Department of Automotive Engineering, Mechanical Faculty, Wrocław University of Science and Technology, Braci Gierymskich 164, 51-640 Wrocław, Poland; konrad.krakowian@pwr.edu.pl (K.K.); maciej.zawislak@pwr.edu.pl (M.Z.); radoslaw.wlostowski@pwr.edu.pl (R.W.); 3 Crowd Sp. z o.o. Sp. kom., Gwiaździsta 10/10, 53-413 Wrocław, Poland; j.cebula@crowd.pl

**Keywords:** vehicle passive safety, sled test, recline seat, autonomous vehicle, ATD crash test dummy

## Abstract

Presently, most passive safety tests are performed with a precisely specified seat position and carefully seated ATD (anthropomorphic test device) dummies. Facing the development of autonomous vehicles, as well as the need for safety verification during crashes with various seat positions such research is even more urgently needed. Apart from the numerical environment, the existing testing equipment is not validated to perform such an investigation. For example, ATDs are not validated for nonstandard seatback positions, and the most accurate method of such research is volunteer tests. The study presented here was performed on a sled test rig utilizing a 50cc Hybrid III dummy according to a full factorial experiment. In addition, input factors were selected in order to verify a safe test condition for surrogate testing. The measured value was head acceleration, which was used for calculation of a head injury criterion. What was found was an optimal seat angle −117°—at which the head injury criteria had the lowest represented value. Moreover, preliminary body dynamics showed a danger of whiplash occurrence for occupants in a fully-reclined seat.

## 1. Introduction

Most passive safety tests are performed with a specifically determined position of the seat, and the ATD (anthropomorphic test device) dummies are carefully seated in order to ensure the repeatability of the tests. However, facing the development of smart vehicles which, in theory, requires minimal driver input, allowing various positions of the seat, an extensive investigation into this matter is justified [1,2,3,4,5]. Presently, the subject of nonstandard seating positions has been relatively rarely investigated. Furthermore, even if there is research about reclined seats, they are mostly in the range accessible to the driver. A fully-reclined seat, as, for example, a sleeping passenger, is nearly always omitted. Such investigations are performed almost exclusively in the simulative environment.

Seat position tendencies depend mainly on the age of the occupants and whether the front or rear seats are considered [1,6,7,8,9,10]. It has been found that a partially reclined seat is pre-dominant while a full reclined position constitutes a small proportion of cases. Moreover, such a position was found predominantly in young men. This, of course, has its impact on the style of driving and, for example, the likelihood of seatbelt use. In consequence, the odds of mortality were the greatest for the fully-reclined seatback. However, a partially reclined seatback, although rated with lower mortality, is represented by a larger population, therefore it represents a significant health concern [7,8,11]. The most severe and commonly investigated factor occurring in a frontal crash with a reclined seat is submarining—i.e., the occupant’s hips sliding under the lap belt, restraining the body at the abdomen [12,13,14]. Submarining causes severe injuries to the lumbar spine and internal organs [15,16,17,18,19]. Accident data analysis shows that the submarining phenomenon is the reason for many fatalities and serious injuries during a frontal impact [20,21,22]. It is claimed that this is due to the low performance of the restraining system securing the occupant’s pelvis [23]. According to [24], submarining occurs when there is no lap belt contact with the iliac crest, the seat cushion allows rotation of the pelvis beneath the lap belt, and no normal friction forces between the lap belt and the iliac crest. Based on a literature survey, it is apparent that such tests are mainly conducted in the numerical environment. Furthermore, the models which predict the injury risk of reclined passengers are limited by the lack of reference data with which to assess fidelity, particularly in terms of the pelvis and lumbar spine motion [25]. The numerical environments for crash simulation provide many models of high biofidelity (e.g., GHBMC-D, GHBMC-S, THUMS), but it appears that the same scenario gives different out-comes for different human body models (HBM) [26]. The main issue is that the Hybrid III ATD is not validated for reclined posture. For example, a standard 50 cc Hybrid III has a constant, unchangeable angle between the thighs and torso. Hence, a numerical investigation must be validated based on volunteer tests (which must be conducted at low speed) or with the aid of postmortem human surrogates—PMHS (various and unpredictable body postures and body reactions to the forces caused by the crash pulse), but there are insufficient data regarding this matter [26,27,28]. In general, based on the worldwide literature, a more reclined position is associated with higher lumbar spine forces, as well as increased possibility of submarining. Therefore, in investigating nonstandard seat positions, the main emphasis is given to the lumbar spine and pelvis [25,29,30]. The occurrence of submarining can be suspected but, apart from the obvious situations related to improper seatbelt–occupant relation, cannot be guaranteed. Submarining depends on the position of the lap belt and the centre of gravity of the pelvis. Hence, as claimed by [31], it can be caused by the anchorage locations or the wearing of a coat.

The dynamics and kinematics of occupants during a crash varies depending on the body posture [32,33,34]. This has a reflection in reclined position crashes. For example, according to [35,36,37], small females were prone to submarining. Moreover, according to [38,39] the pelvis angle was significantly affected by BMI (body mass index). It appears that the pelvis tends to rock backwards with increasing seatback angle. This increases the likelihood of severe injuries, and in the case of obese occupants, the biofidelic model is especially problematic. Hence, improved models of abdominal flesh, and specifically subcutaneous adipose tissue, should be developed prior to employing the human body model (HBM) for crash simulation [40].

Even though the standard ATDs are not validated for reclined seat testing, several attempts to counter this issue have been made. In order to examine the force on the lumbar spine, as well as submarining occurrence, the dummies were equipped with additional, deformable objects at their abdomen. The hybrid III family of dummies can be equipped with a Styrofoam insert which is meant to assess the occurrence of submarining and its risk of injury [41]. A similar addition for child dummies was proposed by [42]. Additionally, the authors validated a numerical model of a child dummy in terms of submarining detection and nonstandard seat positioning body kinematics. Based on their numerical investigation performed on the validated dummy model, it was claimed that dummy knee excursion, torso rotation angle, and the difference between head and knee excursions were good predictors for submarining. It was also found that restraint system design, in particular D-ring height, and the seat coefficient of friction may present an opposing effect of head and abdomen injury risk [8,43].

Due to the severity of the injuries of a reclined occupant, a specific attempt to mitigate this phenomenon was established. Most often this is completed by altering the construction of the seats or installing specifically designed add-ons—for example, alteration of the seat cushion or changing its angle, belt pretensioners, or altering the body kinetics by adding a knee bolster [44,45,46,47,48]. However, inasmuch as those add-ons mitigate the submarining itself, it was proven to be ineffective in terms of overall passive safety [49]. For example, pretensioners increase the belt force during the tension phase, which may lead to abdominal injuries, and when the seat is reclined, it also causes the belt to come closer to the neck, resulting in undesirable chest loading [44,50]. On the other hand, increasing the cushion angle reduces the forward motion and therefore reduces the risk of injures due to lap belt loading [51].

It was found that submarining can be almost entirely avoided by reducing the de-formation of the seat cushion [52,53]. However, a rigid seat constitutes a significant reduction in driving comfort, which ultimately excludes this countermeasure from possible solutions. Nowadays, drivers and passengers tend to use additions that increase the comfort of driving (additional cushion at the back, load limiters, etc.). It has been found, however, that such devices change the body posture, and their excessive usage increases the likelihood of injuries during a frontal impact [54,55]. For example, it was found that with excessive use of such devices, the likelihood of submarining increases as well as the sternal deflection [56].

Injuries of the lumbar spine and the submarining phenomenon are relatively well recognized in the literature. Many researchers have investigated this phenomenon, focusing on the injuries resulting from a reclined seat. However, there are very few papers focusing on the body dynamics of the reclined seat. Different belt performance in terms of restraining an occupant and altered body dynamics can lead to ineffective airbag performance, for instance [57,58,59]. Greater head impact is achieved when submarining occurs, due to greater distance to the airbag [60]. Therefore, the overall body dynamics cannot be neglected by the cost of only the submarining or lumbar spine injuries. The sled test has shown that the distance between the seatbelt anchor and ATD hip is associated with a decrease in head injury criteria (HIC) and an increase in sternal deflection [18,56]. A volunteer test with reclined seats has proven that, apart from the altered body dynamics, reclined occupants suffer larger soft tissue deformation [28]. This could be mitigated by using belt pretensioners, but this would be achieved at the cost of greater axial spine loading [61].

Taking all the above into consideration, the aim of the study was to investigate the influence of the seatback position on an acceleration measured at the dummies head. This acceleration was further used for the evaluation of head injuries predictors (i.e., HIC).

## 2. Materials and Methods

The entire experiment was performed in the laboratory of Vehicle Dynamics and Safety located in a Research Complex GEO-3EM ENERGY ECOLOGY EDUCATION. The investigation of body dynamics with respect to the various seatback inclination was per-formed on a sled rig for component testing. For this test, a standard seat, fixed on rails was used. The seat cushion location was not changed throughout this study—only the position of the seatback was altered. During each test, a standard Hybrid III 50cc ATD was used.

**Figure 1:**
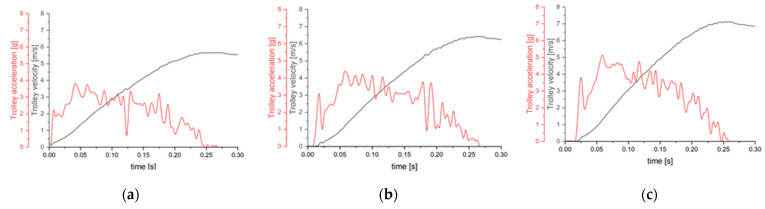
Crash pulses: (**a**) crash pulse 3 [g]; (**b**) crash pulse 4 [g]; (**c**) crash pulse 5 [g].

The tests were recorded with a Phantom Veo 410L high-speed camera. Every test was recorded at 5000 FPS with the maximum available resolution (800 × 1280). The experiment consisted of verifying two variables—i.e., seatback reclination and the crash pulse. The seatback angle ranged from a fully straight comfortable position to fully-reclined. Considering the preliminary nature of this research, as well as the fact that it is to be repeated with volunteers, the generated pulses were relatively low. In general, the volunteer tests are performed in the range of 2.5–5 g. For this reason, the crash pulse was approximated to 3–5 g. Greater crash pulse can constitute a hazard for the human body [16,28]. The values in seatback angle and crash pulse acceleration, as well as the corresponding change of velocity, are shown in Table 1. The crash pulses with the trolley acceleration and velocity with respect to time are shown in Figure 1.

**Figure 2:**
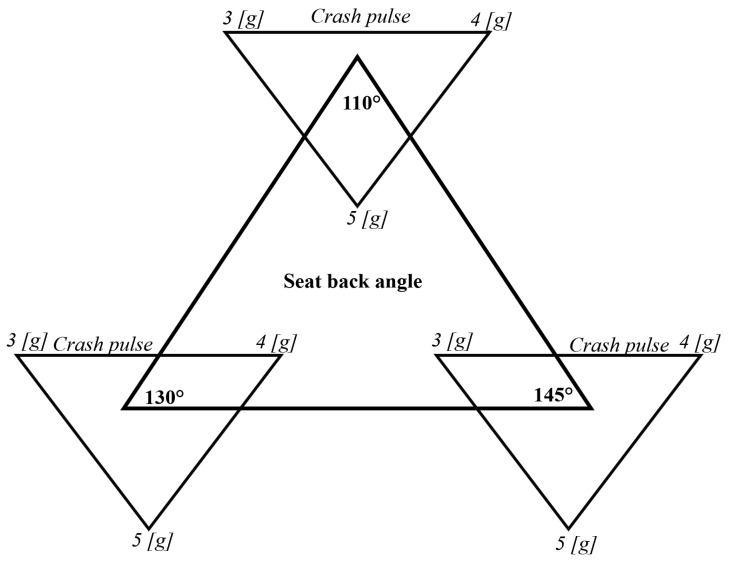
Schematic representation of the design of experiment.

The experiment was performed in a full factorial regime, in accordance with the schematic drawing depicted in Figure 2. In other words, the experiment was performed with each crash pulse on every seatback angle. 

Although the overall movement of the body was considered, the highest emphasis was given to the head dynamics. Therefore, the dummy was equipped with an Endevco piezoresistive accelerometer (model type: 7264C-2KTZ-2-396) measuring head acceleration in X, Y, and Z directions. The resultant acceleration is considered only for the calculation of the injury criteria. The most important direction of acceleration is X (parallel to the trolley motion). This is because the resultant value of acceleration would not contain negative acceleration which, in the case of this study, constitutes backward motion of the head (direction opposite to the trolley motion). The negative acceleration is not within the time limit required for HIC, therefore for calculation of those values, the resultant acceleration filtered with CFC 1000 was used [62]. The sample rate was set to 15 kHz. The acceleration taken was used to calculate the head injury criterion (HIC). Additionally, the duration of the peak was determined.

**Figure 3:**
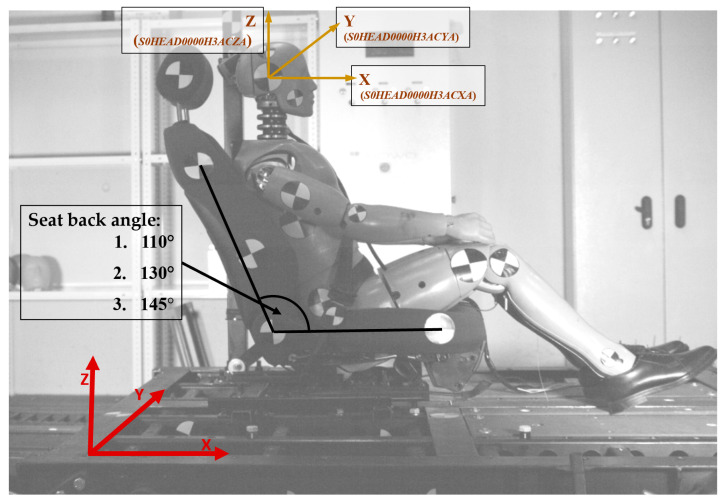
The test station prepared for the tests. The general coordinate system (depicted in red); accelerometers location with channels named according to ISO/TS 13,499 (depicted in gold); seatback angle considered in the experiments (depicted in black).

During the experiment, it was decided not to use any foot constraints because it does not have a significant impact on upper body dynamics, nor on submarining [63]. It was, therefore, considered an unnecessary device in the present investigation. The entire test station is presented in Figure 3.

## 3. Results

**Figure 4:**
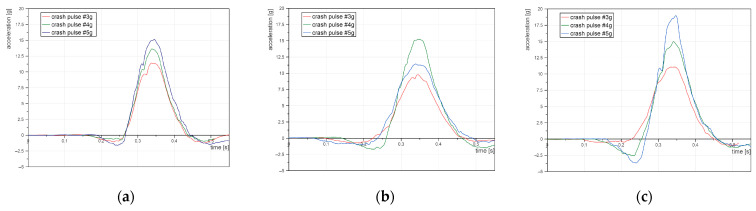
Head acceleration of the dummy: (**a**) seatback angle 110°; (**b**) seatback angle 130°; (**c**) seatback angle 145°.

The results indicate that seat inclination has a significant impact on head acceleration and duration of the acceleration peak and, therefore, on potential head injuries. Comparing the head accelerations achieved with seat inclinations of 110° and 130°, it appears that the greater inclination leads to a lower risk of injury (Figure 4). Not only are the peaks of acceleration similar, but also at 130°, the signals are wider, which represents a longer duration of the acceleration. In consequence, such a position appears to be more beneficial. However, with a fully-reclined seat the head acceleration has the highest values. Furthermore, such movement is enhanced by negative acceleration, which represents backwards head movement.

**Figure 5:**
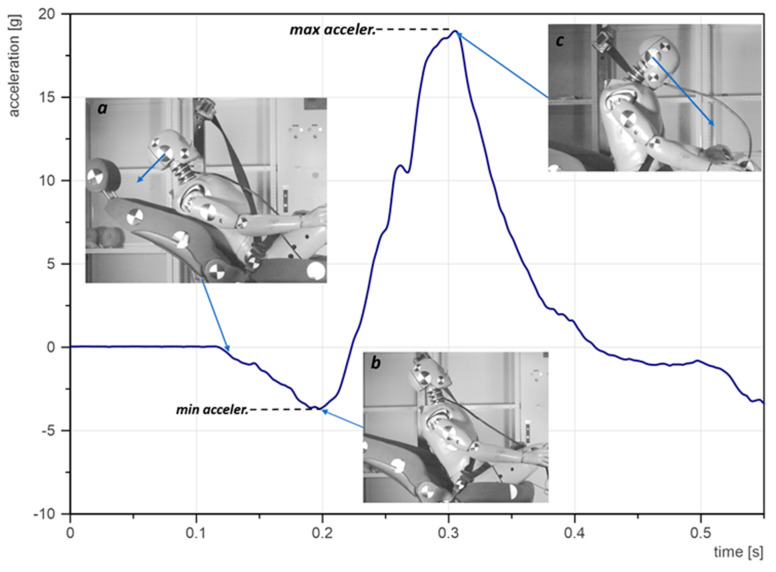
Explanation of the hat acceleration signal: (**a**) backwards motion of the head provoking a negative acceleration; (**b**) change of heads direction of motion; (**c**) forward motion of head provoking the pick of acceleration.

Apart from the obvious head acceleration, the seatback angle causes greater negative acceleration. An analysis of high-speed records revealed that such a tendency is due to seatbelt action. The position of the pelvis on the seat with the fully-reclined seat is farther away from the lap belt. In consequence, at the initial part of the crash pulse, the head and the torso of the dummy lose contact with the head restraints and the seatback, respectively. As can be seen in Figure 5, the hips are then blocked by the belt and the dummy follows the motion of the trolley, however at this point the dummy’s head experiences negative acceleration because the torso propagates to the vertical position (Figure 5a). When the torso meets the shoulder belt, the direction of acceleration is reversed (Figure 5b). Afterwards, the main peak of acceleration occurs, which represents head forward motion. The backwards head motion and, therefore, deceleration had a long duration and relatively low peak, which was not hazardous, however, it enlarges the main peak of acceleration. In other words, the main peak of acceleration was enclosed between the maximum acceleration and minimum acceleration. The more reclined the seat, the higher deceleration and, therefore, the higher the overall acceleration of the head. Moreover, such backwards and forward head motion usually causes whiplash injuries, but this type of injury was not under consideration during these studies.

The entire analysis requires considering both input factors simultaneously (i.e., crash pulse and seat angle). Considering the input factors individually would be impractical. The higher the crash pulse, the higher the value of acceleration. On the other hand, it appears that the seat position changes the overall outcome of the head acceleration. The signal of head acceleration was a basis for determining the standard safety coefficient, Head Injury Criterion (HIC), as well as the average acceleration and the duration of the acceleration. Additionally, the minimal and maximal value of the acceleration signal was measured, the sum of which was the total acceleration which is experienced by the head of the dummy. The entire set of results is shown in Table 2. These results were used to evaluate the response surfaces representing the relation between the input factors and selected property.

**Figure 6:**
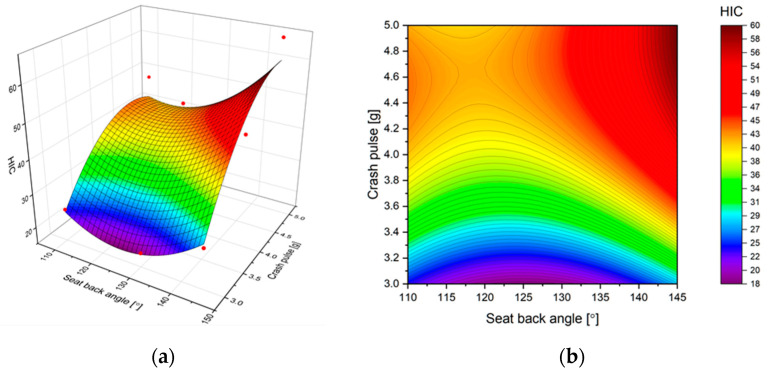
Predicted (estimated) response for HIC: (**a**) fitted response surface; (**b**) fitted response profile.

The 2D and 3D response surfaces of the HIC with respect to the input factors are shown in Figure 6. The red dots on 3D response surfaces represent the measurement points. In general, as expected, the higher the crash pulse, the higher the head acceleration and, hence, the higher the value of HIC. However, this is not the case for the angle of the seatback. It appeared that in almost all response surfaces, there is a saddle point that indicates that there is an optimal value of the angle. For example, considering HIC as an indicator, its value decreases with increasing seatback angle until 117° and increases afterwards. Further increases of seatback angle cause more severe consequences of the crash.

**Figure 7:**
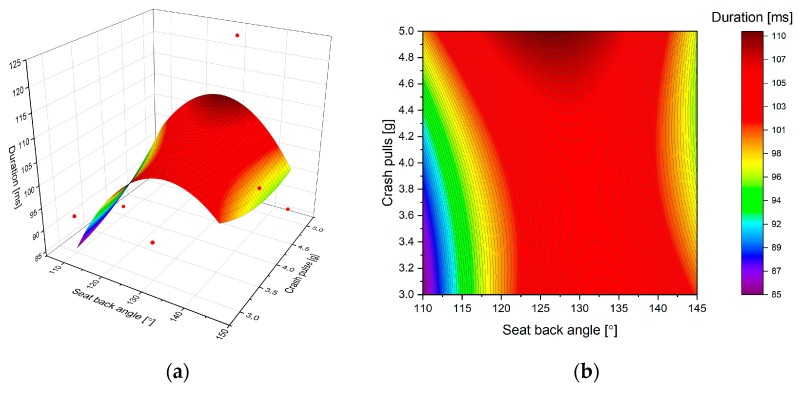
Predicted (estimated) response for the duration of acceleration: (**a**) fitted response surface; (**b**) fitted response profile.

The reason for a saddle point in HIC is the duration of the acceleration peak, which increases for increasing seatback angle (see Figure 7). Moreover, it appears the value of duration does not change significantly for various crash pulses. Such a tendency justifies the need to perform a further, in-depth kinematic study of the dummy at various seat positions.

**Figure 8:**
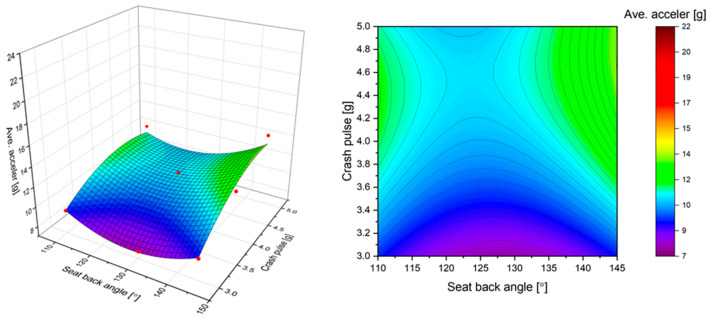

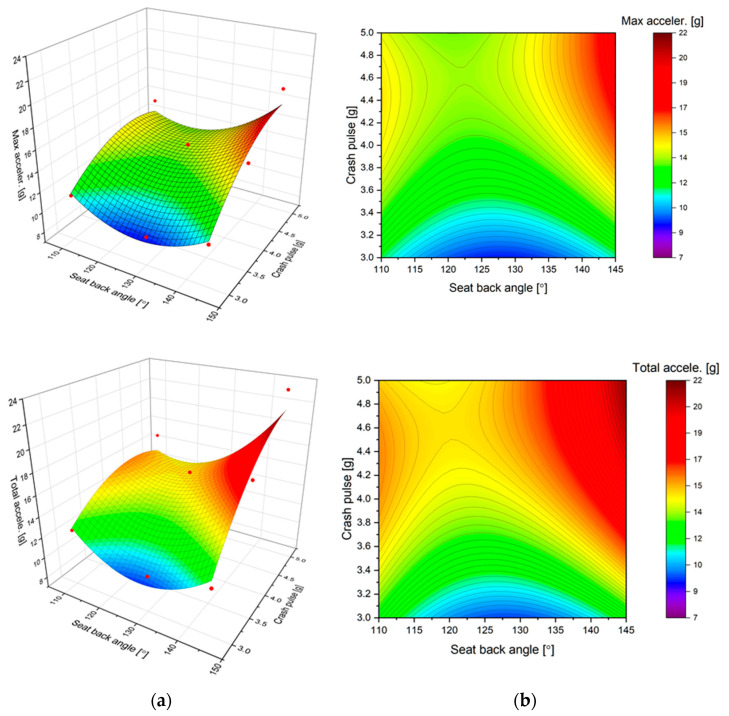
Predicted (estimated) response for acceleration of the dummy’s head: (**a**) fitted response surface; (**b**) fitted response profile.

A comparison of average, maximal, and total acceleration (explained in Figure 5) is shown in Figure 8. The duration of the acceleration pulse affects its average value. The average acceleration calculated with the HIC represents the lowest value. This is because it is calculated over the range of peak duration. In consequence, the peak of acceleration, with no respect to the duration, represents a higher value. The mean difference between the measured peak and calculated average acceleration is 26%, whereas the highest differences exist at an angle of 145° and a crash pulse of 5 g. Similarly, the highest value acceleration was found when the negative acceleration was included in overall consideration. This is especially visible for a fully-reclined seat. In such a case, the head of the dummy experienced the highest increase in acceleration. The mean difference between the total acceleration and the average acceleration reaches 39% but for a fully-reclined seat and the highest crash pulse, the difference is 63%.

This, of course, increases the danger of head injury, but most importantly it is expected to cause a neck injury. In general, the worldwide literature rather poorly describes neck injuries of a frontal crash with occupants sitting on a recline seat. Mainly the lumber spine and submarining are considered.

**Figure 9:**
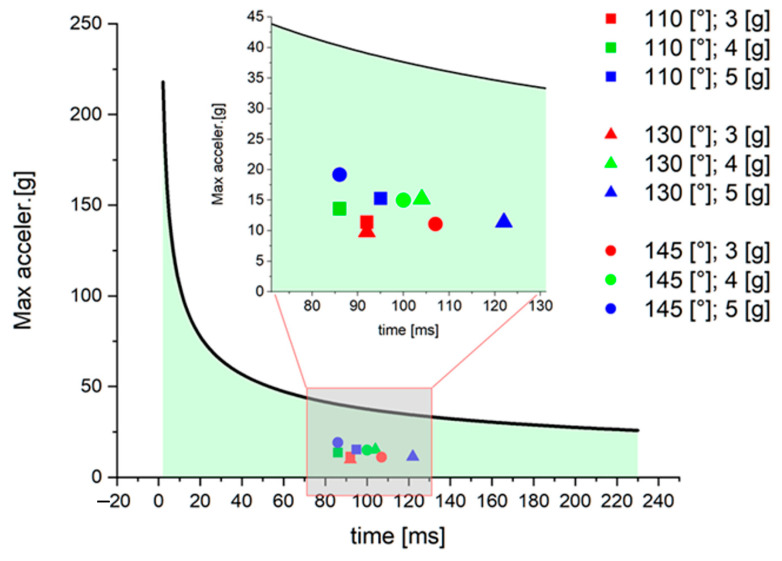
Maximal head acceleration on a Wayne State Tolerance Curve.

The head acceleration presented in these studies was not life-threatening. Maximal acceleration for each experiment run was located on a Wayne State Tolerance Curve (WSTC) in order to verify the severity of the simulated crash (see Figure 9). It appeared that none of the accelerations represent a danger of head injuries. Inasmuch as the duration and acceleration differ by seat position and crash pulse, all of them are in the safe region. Furthermore, it cannot be categorically stated that one result is more severe than another. One could argue whether the acceleration at 145°; 5 [g] represents the highest danger due to the value of acceleration or the test at 130°; 5 [g] due to its longest duration. Nevertheless, all the accelerations are not likely to be life-threatening in terms of head injuries.

## 4. Discussion

The worldwide literature is relatively poor in terms of reclined seat influence on body dynamics. Mostly submarining and lumbar spine injuries are investigated. The present studies provide information on the difference in head performance for various seatback positions. The duration of the acceleration peak can be used to predict the appropriate operation of a restrain systems, such as an airbag or seatbelt design [22,34,45,58,60]. Furthermore, present studies constitute the inside of a safe environment for volunteer testing on this matter.

Nowadays, the various seat positions while driving are gaining greater importance due to the introduction of autonomous vehicles. Most of the research completed in this matter is only conducted in the numerical environment and is rarely verified with experiments using a dummy, volunteer, or postmortem human surrogate (PMHS) [27]. Generally, the research focuses on the submarining effect and lumbar spine injury. The head kinetics for reclined seat positions were considered in [61] where the authors stated that at high speeds the submarining of the model dummy occurred, which caused increased head impact velocity due to greater distance from the airbag. The authors suggested that the head impact severity could be mitigated with an appropriate seatback angle. The research described in this paper is in conjunction with our findings. Based on our research, it appeared, however, that the relationship between the head movement caused by the change of vehicle velocity and the seat position is not linear and there is a local optimum which does not constitute the far-right position.

The dummy kinetics, and, therefore, head acceleration, are originated from the position of the dummy’s pelvis and hips on the seat. The various angles of the seatback affect the positioning of the dummy. The seatback moves behind the pelvis as it reclines [38]. Additionally, as was proven in the present study, negative head acceleration occurs due to backwards head movement, which is reversed only when the hips are stopped by the lap belt. Greater distance between hips and the belts generates longer free-movement of the dummy during which the head is no longer supported by the head restraint. Furthermore, the hips experience stronger hits by the belt [64]. This, in turn, causes dynamic backwards movement of the dummy’s head, increasing the total acceleration.

Dummies are positioned differently than human passengers, which affect the overall results. In general, according to [65], the lap belt of a passenger is typically much farther from the dummy’s pelvis. Furthermore, the design of the dummies introduces an error to the overall investigation. For example, the weight distribution between the thighs and the pelvises of the dummies is usually different from a passenger’s, and also the stiffness of the abatement or skeletal frame [66]. Therefore, in order to fully analyse the various seat positions, surrogate testing should be performed. The thresholds presented here suggest that such tests are not hazardous for humans in terms of head acceleration and the lack of submarining. However, a significant head motion backwards and forwards was observed especially for a fully-reclined seat. Such movement can cause a severe whiplash injury, which was not under consideration during these studies. Additionally, the research of reclined seat positions focusses primarily on the pelvis and lumbar spine injuries due to submarining, not on neck injuries, hence, a similar study which includes dummy kinematics and neck injuries for various seat positions is justified. The determination of a neck injury criterion was not in the scope of this study mainly due to the fact that such significant head and neck behaviour was not anticipated. The literature-based knowledge focuses only on lumbar spine injuries and submarining. Nowadays, there are a variety of means to mitigate submarining and, hence, mitigate the severity of the lumbar spine injury [8,16,49,54,59,67]. However, as it appeared during this study, even if submarining does not occur, the body kinetics on a reclined seat can be hazardous in terms of neck injuries. Therefore, further in-depth research consisting of a body kinetics analysis on a reclined seat, as well as a neck injury criterion analysis is justified.

## 5. Conclusions

The research performed here was completed firstly to evaluate the relationship between the crash pulse and the seatback angle on human performance. What was taken into account was the acceleration in the dummy’s head, which was further used for the determination of head injury criterion. The response surfaces developed based on acceleration and HIC measures with respect to the crash pulse and seatback angle revealed the nonlinearity of such dependence. A saddle point was found in HIC, and, therefore, accelerations, which suggests that there is an optimal seat position. It is speculated that the reason for such a change tendency is the duration of head acceleration. Furthermore, it was found that the acceleration and, therefore, the head injury criterion increases with increasing angle of the seatback. There is an optimal value of seat reclination. With the experiment regime, the optimal seat angle in terms of head acceleration was 117°. Inasmuch as head accelerations determined during this research do not constitute a danger in terms of head injuries, a significant backward and forward motion of the head was detected during the test with a reclined seat. This phenomenon could not be avoided due to the altered position of the dummy’s pelvis on the seat. In consequence, even when the dummy’s head rested on a restraint prior to the crash pulse, there was a longer time required for the pelvis of the dummy to reach the lap belt. During this time, the head travelled backwards. This motion was reversed only when the torso of the dummy was blocked by the shoulder belt. Such motion is a potential reason for neck injuries.

## Figures and Tables

**Table 1:** Input factors of the experiment.

Input Factors	Min Value	Centre Value	Max Value
Seat back angle	110°	130°	145°
Crash pulse	3 (±0.3) g	4 (±0.3) g	5 (±0.3) g
Crash pulse ΔV	5 (±0.4) m/s	6 (±0.4) m/s	7 (±0.4) m/s

**Table 2:** The accelerations for all crash pulses as well as the acceleration peak duration and HIC.

Seat Back Angle	Crash Pulse (g)	HIC	Duration (ms)	Ave. Acceler. (g)	Min Acceler. (g)	Max Acceler. (g)	Total Acceler. (g)
110°	3	24.6	92.5	9.3	−1.1	11.4	12.5
	4	34.1	86.3	10.9	−0.6	13.6	14.2
	5	48.0	95.0	12.1	−1.4	15.3	16.7
130°	3	19.1	92.5	7.7	−0.7	9.8	10.5
	4	52.0	104.5	12.0	−1.7	15.2	16.9
	5	32.6	123.5	9.3	−1.0	11.4	12.4
145°	3	26.9	106.9	9.14	−0.5	11.1	11.5
	4	47.5	99.9	11.78	−2.6	15.0	17.5
	5	66.5	86.8	14.25	−4.0	19.2	23.2

## Data Availability

The data presented in this study are available on request from the corresponding author. The data are not publicly available due to privacy.
